# Working Cannula-Based Endoscopic Foraminoplasty: A Technical Note

**DOI:** 10.1155/2018/4749560

**Published:** 2018-12-23

**Authors:** Suxi Gu, Kedong Hou, Wei Jian, Jianwei Du, Songhua Xiao, Xifeng Zhang

**Affiliations:** ^1^Orthopedic Department, Beijing Tsinghua Changgung Hospital, School of Clinical Medicine, Tsinghua University, Beijing 102248, China; ^2^Orthopedic Department, Beijing Friendship Hospital Pinggu Campus, Capital Medical University, Beijing 101200, China; ^3^Orthopedic Department, Beijing PLA 309 Hospital, Beijing 100091, China; ^4^Orthopedic Department, Yangzhou No. 1 People's Hospital, Yangzhou, Jiangsu Province 225001, China; ^5^Orthopedic Department, PLA General Hospital, Beijing 100853, China

## Abstract

**Purpose:**

Percutaneous endoscopic lumbar discectomy (PELD) is a minimally invasive disc surgery that can be performed under local anesthesia and requires only an eight-mm skin incision. For the patients with lumbar foraminal stenosis, the migrated disc is difficult to remove with a simple transforaminal approach. In such cases, the foraminoplasty techniques can be used. However, obtaining efficient foramen enlargement while minimizing radiation exposure and protecting the nerves can be challenging.

**Methods:**

In this study, we propose a new technique called the Kiss-Hug maneuver. Under endoscopic viewing, we used the bevel tip of a working cannula as a bone reamer to enlarge the foramen. This allowed us to efficiently enlarge the lumbar foramen endoscopically without the redundancy and complications associated with reamers or trephines.

**Results:**

Details of the four steps of the Kiss-Hug maneuver are reported along with adverse events. The advantages of this new technique include minimizing radiation exposure to both the surgeon and the patient and decreasing the overall operation time.

**Conclusion:**

The endoscopic Kiss-Hug maneuver is a useful and reliable foraminoplasty technique that can enhance the efficiency of foraminoplasty while ensuring patient safety and reducing radiation exposure.

## 1. Introduction

Although open lumbar discectomy is the gold standard surgical technique for lumbar disc herniation, iatrogenic damage on the facet joints and other paraspinal structures along with reduced disc height, segmental instability, and retrolisthesis may become a problem [1, 2]. Therefore, percutaneous endoscopic lumbar discectomy (PELD)'s transforaminal approach is gaining recognition. It has many advantages including reduced paraspinal muscle trauma, minimal postoperative instability, and a smaller surgical wound [3–5]. Transforaminal approach provides easy access to the entirety of the bulging or calcified disc, the inferior facet, and the front of the laminae [6, 7]. The enlargement of the target foramina provides direct access to the lateral foraminal canal and direct visualization of the superior face, the main culprit in lateral spinal canal [7–9].

In patients with foraminal bony stenosis, osteophytes on the substantial superior articular process (SAP) are challenging to remove. Before the operation, patients get a prone position on a radiolucent operating table. Under fluoroscopic guidance, an 18-G needle is inserted. The target position of the needle tip just prior to puncture of the disc is on the posterior vertebral body line on the lateral C-arm view, and on the medial pedicular line on the anteroposterior view. This should correspond to the safe triangle in the axillary area between the exiting and traversing nerve root. In patients with disc fragment migrations, the ideal needle position is difficult to achieve [10–13]. Foraminoplastic procedures, such as removal of SAP osteophytes to widen the lumbar foramen and removal of parts of the facet and ligamentous tissue surrounding the foramen, are sometimes required to allow the endoscope to enter [7, 10–15]. Multiple studies [7, 9] have indicated that medial access to Kambin's triangle by foraminoplasty provides safer access to the intraforaminal space and makes it possible to prevent exiting nerve injury.

Technically, bone reamers or trephines can quickly cut off hypertrophied SAP or osteophytes [7]. However, as blind techniques, these tools have inherent disadvantages. C-arm-guided foraminoplasty may cause unintended multifluoroscopic exposure, inadequate bone removal, bleeding, significant bony structure removal causing lumbar instability, and even sensitive neural damage [16–20]. To address these concerns, specialized tools for endoscopic foraminoplasty have been developed, such as endoscopic drills, high-speed diamond and articulated burrs, punches, forceps, osteotomes, and the straight- and side-firing Holmium-YAG laser [15, 21]. Unfortunately, these techniques can be less efficient and more time-consuming in cases of severe bony stenosis. Therefore, a more efficient endoscopic method to enlarge the stenotic foramen is needed.

We propose a Kiss-Hug maneuver to efficiently and safely decompress foraminal stenosis, utilizing one of the fundamental tools in the PELD procedure: the working cannula. This technique maximizes the effectiveness of endoscopic decompression while ensuring patient and surgeon safety. In this endoscopic foraminoplasty maneuver, the bevel tip of the working cannula is used as a bone reamer to undercut the SAP without the need for any other specific instrumentation.

## 2. Technical Note

**Figure 1:**
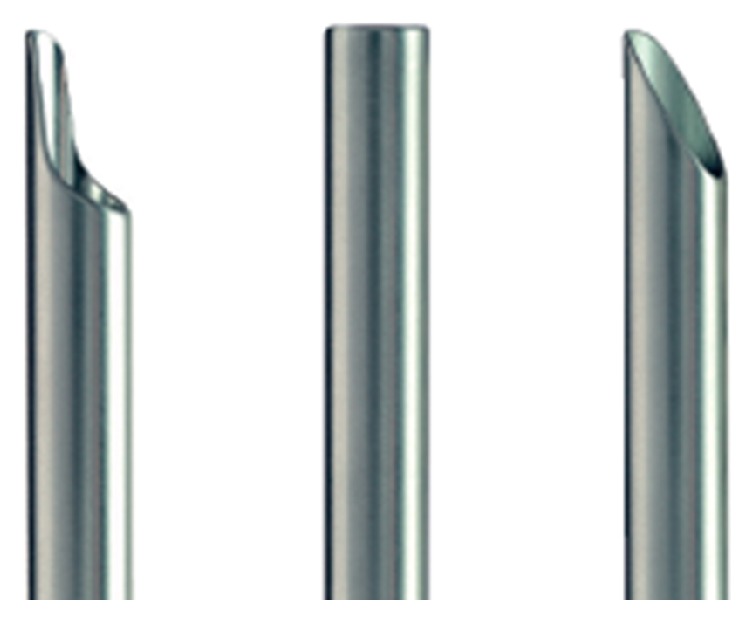
Schematic designs of the working cannula tip. We use a working cannula with the bevel tip (first tip on the right) in the Kiss-Hug procedure.

The working cannula is the only equipment required for the Kiss-Hug technique. Although working cannulas are available in different outer diameters, working lengths, and tip configurations, we chose the working cannula with a bevel (distal oblique) tip to minimize the occupying effect of the surgical equipment [Figure 1]. Because the normal vertical and transverse dimension of the lumbar foramen is only 12–19 mm and 12–14 mm, respectively, any space-occupying pathology or instruments can contribute to the existing foramen stenosis and lead to severe nerve impingement [22].

**Figure 2:**
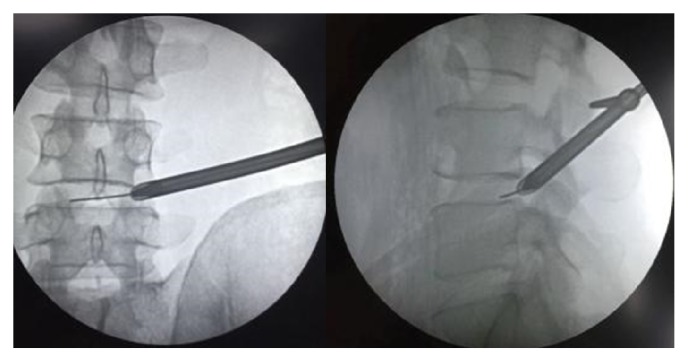
The ideal position of the bevel tip in the working cannula under fluoroscopic anterior posterior view (A) and lateral view (B).

**Figure 3:**
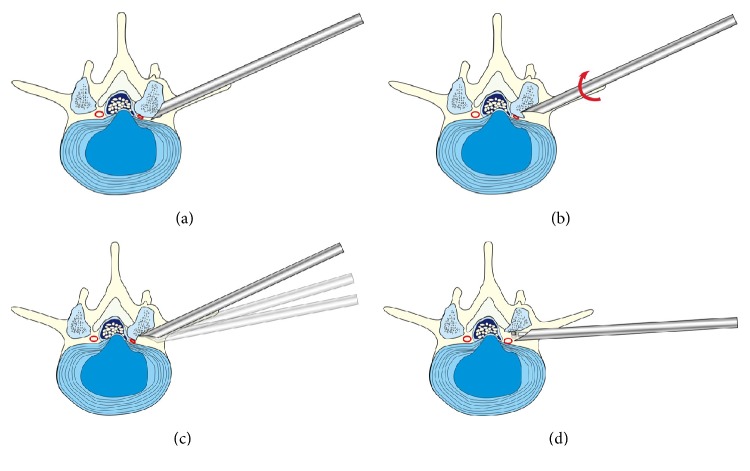
(a) Schematic of the Kiss step: the tip of the working cannula should anchor between the superior articular process (SAP) and the posterior wall of the disc or distal vertebra through the foramen. The bevel side should face upward and dorsally, so that the bevel tip can fit perfectly and securely on the ventral-lateral side of the SAP. (b) Schematic of the Hug step: after identifying the location and morphology of the osteophyte, the surgeon rotates the working cannula to shave off the osteophyte on the superior articular process (SAP), utilizing the bevel tip in a piecemeal fashion. The surgeon must ensure that the working cannula does not advance too far into the spinal canal and that the exiting nerve root is kept outside the protective working cannula. (c) Schematic of the Tilt step: the working cannula can be tilted upward, downward, or leveled to address different pathological requirements until sufficient foramen enlargement has been achieved. The exiting nerve root is particularly vulnerable during this step. Excessive manipulation of the working cannula can cause pressure on the dorsal root ganglion, leading to severe intraoperative pain and postoperative dysesthesia. (d) Schematic of the Finishing step: after shaving off the majority of the osteophyte using Kiss-Hug maneuvers, the opening of the foramen window continues. If necessary, other endoscopic tools such as articulate burrs and side-firing lasers could be used to further remove remnant osseous fragments and thickened ligamentous materials.

A detailed description of a PELD procedure using the Kiss-Hug maneuver is provided as follows. The procedure begins by advancing the working cannula down to the foramen, following the tapered obturator engaged into the foramen. Before introducing endoscopy, the position of the working cannula should be checked under fluoroscopy. The surgeon must ensure that the tip of the working cannula has not advanced beyond the medial-pedicle line in the anterior posterior view and touches the ventral side of SAP in the lateral view [Figure 2]. The tip of the working cannula should anchor between the SAP and the posterior wall of the caudal vertebra or disc (depends on varies anatomy, away from exiting nerve root, touching the upper surface of caudal pedicle) through the foramen [Figure 3(a)]. At this point, the surgeon should confirm that the bevel tip is facing upward and dorsally, so that the tip fits perfectly into the space and is firmly secured on the ventral side of the SAP. Therefore, the working cannula could gently* kiss* the SAP. This position allows the next step in the maneuver to occur without any slipping or shifting.

After the working cannula is anchored in the foramen, the endoscope is introduced through the cannula. A thorough endoscopic exploration of the foraminal space is performed using a bipolar coagulator. The thickened ventral parts of the facet capsule are removed until the ventral part of the SAP is visualized. Once the location and morphology of the osteophyte has been identified, the surgeon can begin the hug maneuver [Figure 3(b)]. Holding the rear-handle, the surgeon rotates the working cannula, applying a moderate amount of force and constant endoscopic control, as if he is* hugging* the osteophyte.

Typically, the surgeon will use a left-handed fingertip grip on the endoscopy to control the direction of the working cannula and to provide constant direct endoscopic viewing [Figure 5]. Using the right hand to hold the rear-handle, the surgeon can rotate the working cannula and apply the shaving force axially, but not vertically. The blunt edge (about one mm thickness) of the bevel tip works as a bone-cutting blade, and the endoscopy itself works as the rotational axis. Under the twisting or rotating movement of the working cannula, the ventral portion of the osteophyte SAP can be shaved into pieces by the bevel tip. The bone chips can be removed along with the endoscopy from the working cannula. After this step, the bevel tip can be pressed onto the SAP again, and additional Kiss-Hug maneuvers can be performed until enough foraminoplasty has been achieved. This maneuver actually poses a lot shear stress to the bevel tip that may lead to damage of the working cannula. Therefore, the extra combination usage of electrical articulated burrs might be helpful.

For better bone cutting and thorough lumbar foramen enlargement, the elasticity of the muscle tissue and the mobility of the lumbar skin can be used to change the positions and directions of the foraminoplasty [Figure 3(c)]. By holding the endoscopy as a direction-controller and using the cannula's bevel tip as a fulcrum, the surgeon can adjust the trajectory inclination of the working cannula [15].

**Figure 4:**
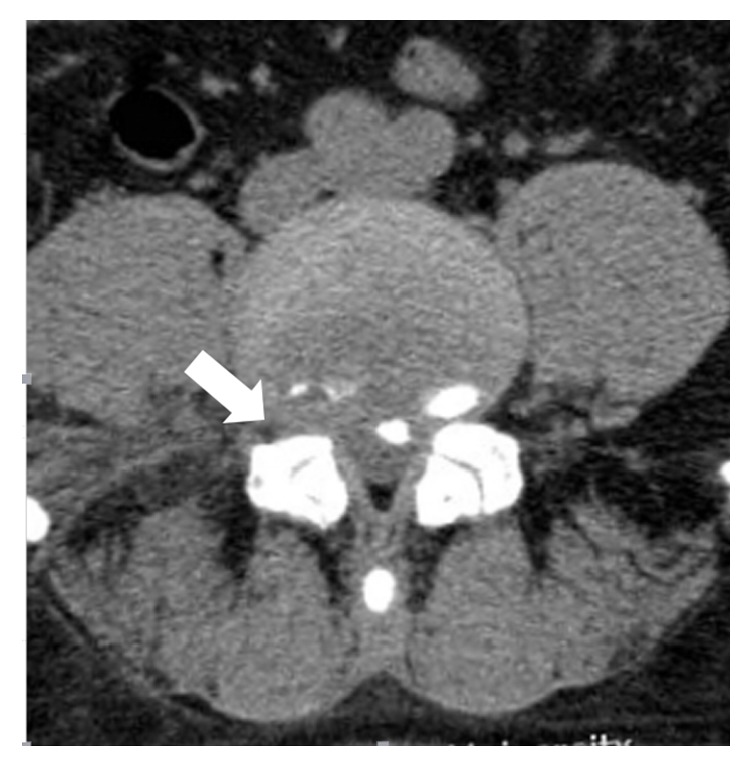
Postop axial CT image (white arrow) shows enlargement of narrowed foramen (compared to contralateral side) and preservement of the facet joint.

**Figure 5:**
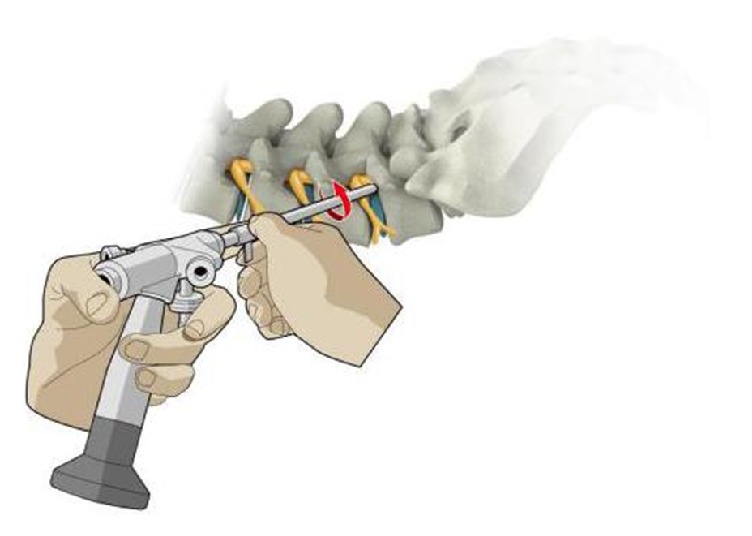
A diagrammatic sketch showing how the endoscopy works as the rotational shaving axis while the surgeon rotates the working cannula. During this procedure, some ligamentous flavum and other foraminal ligaments should be left between the bevel tip and the neurostructure to avoid any nerve damage during the procedure.

Once the target foramen has been adequately enlarged [Figure 4], the rest of the procedure is not different from the conventional technique. Remnant osseous fragments and thickened ligamentous material can be removed using endoscopic forceps, articulating burrs, and coagulators until the epidural space and the dura are visualized [Figure 3(d)]. Finally, the surgeon can completely remove the migrated or sequestered discs.

## 3. Discussion

Foraminoplasty has been reported as a useful surgical strategy in degenerative lumbar foraminal stenosis, in which the nerve root is entrapped in a narrowed foramen [7, 12, 15, 21, 23, 24]. Traditionally, foraminoplasty could be categorized into two classifications: fluoroscopy-dependent or endoscopy-dependent. Many specialized microsurgical tools for foraminal stenosis decompression have been described, ranging from reamers and trephines to endoscopic drills and lasers [13, 25, 26].

Fluoroscopy-dependent tools, such as sequential reamers or trophies, are potent and can rapidly cut off hypertrophied SAP. However, safety is a concern, because sequential reaming can lead to neural injury and accidental bleeding [17–20, 27, 28]. Because of these issues, the procedure must be carefully monitored under fluoroscopy, and the reamer tip should not advance over the medial-pedicle line [21]. This means that both patients and surgeons risk multiple radiation exposures [29, 30]. In contrast, the Kiss-Hug maneuver is conducted under endoscopic guidance, so the amount of fluoroscopic exposure is significantly reduced. Most neural injury incurred during fluoroscopy-dependent foraminoplasty is related to the serrated tip of the bone reamer advancing too far beyond the medial border of the facet joint. As opposed to the bone reamer or the trephine, the distal tip of the working cannula contains no saw structures. The beveled tip is smooth and blunt, and it is not sharp enough to cut through the foraminal ligament. If this maneuver proceeds under general anesthesia, the ligament flavum and the intraforaminal ligament function as an anatomical barrier to prevent neural injury. In the circumstances of local anesthesia, instant feedback from the patient throughout the procedure if he or she is experiencing leg pain might add extra help.

Meanwhile, excellent endoscopic burr systems such as the ultra-thin high-speed drill and the articulated burr [7, 15, 22] could provide safer and more efficient foraminotomy effect than any trephine or bone reamer. The surgeon could accomplish this foraminoplasty under direct endoscopic observation, minimizing neural injury and potential bleeding. However, compared with the fluoroscopy-guided option, expensive additional equipment is needed for these procedures. The Kiss-Hug maneuver utilizes the rotational movement of the working cannula without any other equipment and can remove bony structures more efficiently (Kiss-Hug maneuver takes approximately five to ten seconds) than other methods. Because it is hand-driven, the Kiss-Hug maneuver also avoids the risk of heat-damage to the surrounding spinal nerves that has been reported in other endoscopic procedures [31, 32]. Another issue with endoscopic burr systems is that it is very bulky and difficult to manipulate. Navigating the burr can be challenging; sometimes, the surgeon even needs help from an assistant to hold and manage the equipment. In contrast, the working cannula used for the Kiss-Hug maneuver has a relatively short leverage and can be driven by hand. It provides better force feedback and more precise control than other tools.

One of the advantages of endoscopic foraminoplasty is that it can be individualized for each patient and the specific pathology of the narrowed foramen [33]. Using the elasticity of the surrounding skin and muscle tissue as a fulcrum, the position and direction of the beveled tip can easily be adjusted [13, 33–35]. A surgeon can remove hypertrophied osteophytes from the SAP by hand, particularly the marginal osteophyte that hinders the passage of the working cannula.

Many articles have reported that patients experience a great amount of pain during foraminoplasty, so most surgeons use anesthetics (10–20 ml) on the SAP surface [10, 12, 13, 34–37]. We have not experienced this problem in our practice, perhaps because we routinely coagulate the soft tissue on the SAP's ventral surface before conducting the Kiss-Hug maneuver. During this coagulation step, the sino-vertebral nerve surrounding the foramen may become desensitized.

Another concern in foraminoplasty is the risk of bleeding from an injury to the venous sinus or the bony facet surface. The way we handle this issue in the Kiss-Hug maneuver is not different from other techniques [7, 38, 39]. Most intraoperative bleeding is minimal and spontaneously controlled by compression with the working cannula. Soft tissue bleeding can be managed with a flexible bipolar radiofrequency probe.

Although the Kiss-Hug technique has produced favorable results, some technical limitations remain. Not all kinds of foraminal stenosis can be treated using the Kiss-Hug maneuver, and the indication of Kiss-Hug maneuver is not identical to other endoscopic foraminoplasty tools. For example, a diamond burr is often used for bone removal near important structures, because it is less likely to cause injury, given its delicate drilling capabilities compared to a fluted steel burr [15]. Laser has the potential to remove osteophytes as well as inflamed soft tissue, including hypertrophied capsule, within a narrowed foramen [31]. The best use of the Kiss-Hug maneuver is at the beginning stage of foraminoplasty, so that the surgeon can easily shave off a large amount of bony structure within seconds, enhancing his working efficiency. For deep, localized, and small osteophytes in the stenotic canal, other specialized tools such as the power-articulated burr or the side-firing laser are more appropriate. The Kiss-Hug technique is an alternative surgical option to be considered for foraminoplasty procedure. Meanwhile, the working cannula is not designed to cut any bone tissue; there will be an additional concern for the instrumental failure or breakage in younger patients with stiffer bony structure. When the working cannula hugs the ventral portion of the SAP, the shear stress on the bevel tip might cause it to break. The possibility of tip fracture is higher when undercutting larger pieces of osteophyte. Therefore, we suggest using piecemeal methods. Finally, care should be taken to avoid using the working cannula in a defective or damaged condition, since an articulated drill could accidentally burr the inner surface of the working cannula and cause weak points during the procedure.

## 4. Conclusions

Our experiences indicate that working cannula-based foraminoplasty could be a viable complement to conventional methods of endoscopic foraminoplasty, and it has some competitive advantages over other surgical tools, including better force feedback, higher cutting efficiency, and more precise control.

## Data Availability

The technical data of foraminoplasty used to support the findings of this study are included in the article.

## Supplementary Materials

Supplementary Materials An endoscopic video of working cannula-based foraminoplasty (Kiss-Hug maneuver). We use the bevel tip of a working cannula as a bone reamer to enlarge the foramen.
